# New species in the *Sitalcina sura* species group (Opiliones, Laniatores, Phalangodidae), with evidence for a biogeographic link between California desert canyons and Arizona sky islands

**DOI:** 10.3897/zookeys.586.7832

**Published:** 2016-05-04

**Authors:** Angela DiDomenico, Marshal Hedin

**Affiliations:** 1 Department of Biology, 5500 Campanile Drive, San Diego State University, San Diego, CA 92182-4614, USA

**Keywords:** Species delimitation, plate tectonics, short-range endemism, historical biogeography, Bayes Factor Delimitation, Madro-Tertiary Geoflora

## Abstract

The western United States is home to numerous narrowly endemic harvestman taxa (Arachnida, Opiliones), including members of the genus *Sitalcina* Banks, 1911. *Sitalcina* is comprised of three species groups, including the monospecific *Sitalcina californica* and *Sitalcina lobata* groups, and the *Sitalcina sura* group with eight described species. All species in the *Sitalcina sura* group have very small geographic distributions, with group members distributed like disjunct “beads on a string” from Monterey south to southern California and southeast to the sky-island mountain ranges of southern Arizona. Here, molecular phylogenetic and species delimitation analyses were conducted for all described species in the *Sitalcina sura* group, plus several newly discovered populations. Species trees were reconstructed using multispecies coalescent methods implemented in *BEAST, and species delimitation was accomplished using Bayes Factor Delimitation (BFD). Based on quantitative species delimitation results supported by consideration of morphological characters, two new species (*Sitalcina oasiensis* **sp. n.**, *Sitalcina ubicki* **sp. n.**) are described. We also provide a description of the previously unknown male of *Sitalcina borregoensis* Briggs, 1968. Molecular phylogenetic evidence strongly supports distinctive desert versus coastal clades, with desert canyon taxa from southern California more closely related to Arizona taxa than to geographically proximate California coastal taxa. We hypothesize that southern ancestry and plate tectonics have played a role in the diversification history of this animal lineage, similar to sclerophyllous plant taxa of the Madro-Tertiary Geoflora. Molecular clock analyses for the *Sitalcina sura* group are generally consistent with these hypotheses. We also propose that additional *Sitalcina* species await discovery in the desert canyons of southern California and northern Baja, and the mountains of northwestern mainland Mexico.

## Introduction

Laniatorean harvestmen comprise the majority of Opiliones diversity, with more than 4100 described species (Kury 2013). North temperate Laniatores are typically small (body length usually less than 3 mm), short-legged animals, most often found in sheltered microhabitats such as under rocks and logs. The western United States is home to a diverse laniatorean fauna, with more genera and species than any other region in the Nearctic, making this area a harvestman “hotspot” (e.g., Briggs 1971, Ubick and Briggs 1989, Ubick and Briggs 2008, Derkarabetian and Hedin 2014). Laniatores of the family Phalangodidae are mostly Holarctic in distribution, but particularly diverse in the Nearctic (Ubick 2007). In California there are about 70 described phalangodid species, and this number has been growing with further sampling and use of SEM studies of morphology. Most of the described species have very small geographic distributions, and deserve more evolutionary, biogeographic, and conservation interest than currently afforded (Ubick and Briggs 2008, Emata and Hedin 2016).

**Figure 1.**
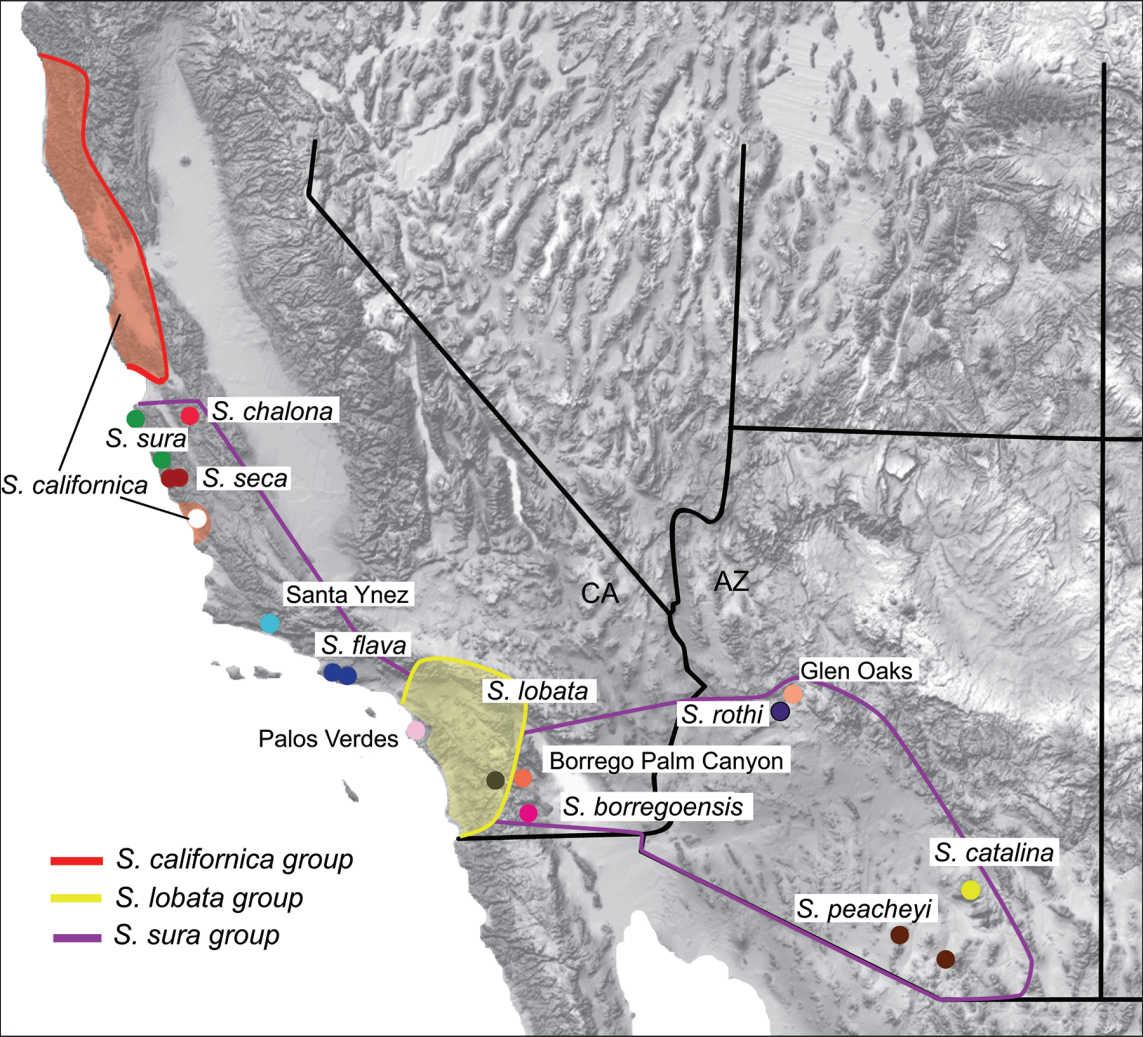
Distribution of *Sitalcina* and geographic sample. Groups include *Sitalcina californica* (1 species), *Sitalcina lobata* (1 species), and *Sitalcina sura* (8 described species, 4 geographically novel populations). Sampled populations indicated by circles. General species distributions follow Ubick and Briggs (2008).

The phalangodid genus *Sitalcina* is comprised of three species groups (Ubick and Briggs 2008), including the monospecific *Sitalcina californica* and *Sitalcina lobata* groups, and the *Sitalcina sura* group which includes eight described species: *Sitalcina sura* Briggs, 1968, *Sitalcina seca* Ubick & Briggs, 2008, *Sitalcina chalona* (Briggs, 1968), *Sitalcina flava* Briggs, 1968, *Sitalcina borregoensis*, *Sitalcina rothi* Ubick & Briggs, 2008, *Sitalcina catalina* Ubick & Briggs, 2008 and *Sitalcina peacheyi* Ubick & Briggs, 2008. With a known distribution from Monterey south to southern California and southeast to the sky-island mountain ranges of southern Arizona (Figure 1), species of the *Sitalcina sura* group occupy a variety of upland habitats. Most of these habitats are dominated by sclerophyllous woody plant taxa (e.g., *Quercus*, Pinyon pine, *Arctostaphylos*, *Ceanothus*, etc.), part of the Madro-Tertiary Geoflora (MTG, Raven and Axelrod 1978, Lancaster and Kay 2013, Baldwin 2014). Exceptions include *Sitalcina sura* from redwood forests, and *Sitalcina borregoensis* from desert canyons, below the elevational level of desert chaparral. Species from the sky-island mountain ranges of southern Arizona occur in mid-elevation Madro-Tertiary habitats, largely below higher elevation conifer forest, but above low desert habitats. In all habitats *Sitalcina sura* group members occupy seemingly similar microhabitats, typically under rocks on shaded north-facing slopes (Ubick and Briggs 2008, Figure 2).

**Figure 2.**
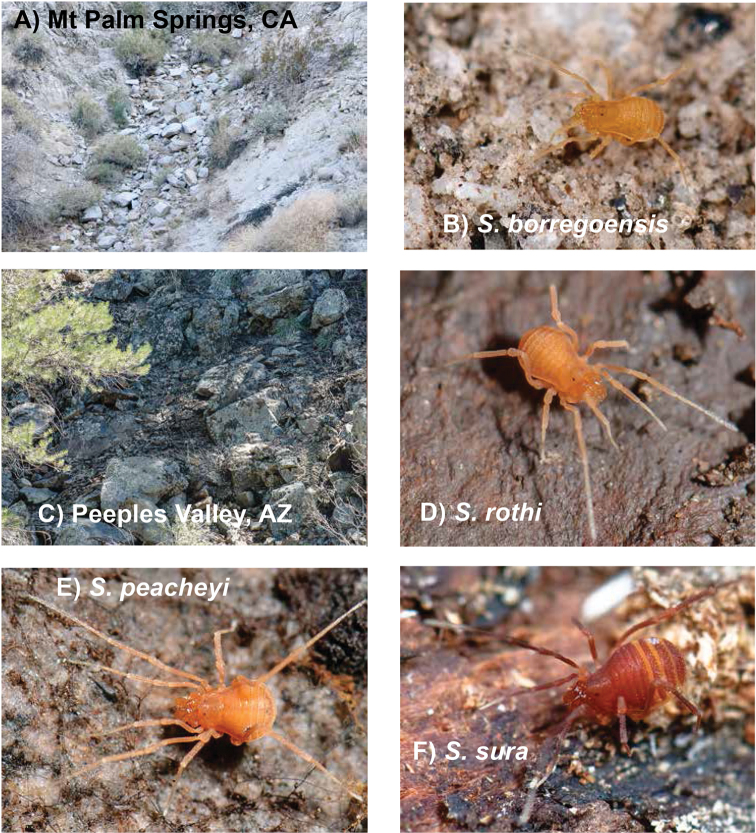
Habitats and live *in situ* specimens. **A** Granite talus at Mt Palm Springs, CA – creosote + ocotillo + bursage habitat **B** live *Sitalcina borregoensis* from Mt Palm Springs **C** Volcanic talus at Peeples Valley, AZ – pinyon, juniper habitat **D** live *Sitalcina rothi* from Peeples Valley **E** live *Sitalcina peacheyi* from Madera Canyon, AZ **F** live *Sitalcina sura* from Palo Colorado Road, CA. Specimen images not at same scale.

Because of apparent limited dispersal abilities and microhabitat specificity, extreme population genetic structuring and divergence can be expected in *Sitalcina*, as is observed in other low vagility Laniatores (e.g., Thomas and Hedin 2008, Hedin and Thomas 2010, Derkarabetian et al. 2011, Starrett et al. 2016, Emata and Hedin 2016). As such, some currently described species with naturally fragmented distributions (e.g., *Sitalcina peacheyi* on isolated mountaintops and in caves) may actually comprise multiple cryptic species. Also, the geographic sampling of previous studies may have missed unique species. Finally, members of the *Sitalcina sura* group are very similar in somatic morphology, likely due to niche conservatism (Wiens and Graham 2005, Keith and Hedin 2012). Overall, the *Sitalcina sura* group has the potential to include both newly discovered and cryptic species.

A variety of objective species delimitation methods have been developed in recent years (e.g., Yang and Rannala 2010, Rannala and Yang 2013, Grummer et al. 2014). Several of these species delimitation methods are founded on the multispecies coalescent model, utilizing multilocus genetic data (summarized in Fujita et al. 2012, Carstens et al. 2013). The probable existence of morphologically cryptic species in *Sitalcina* makes the use of such genetic methods attractive. Also, because *Sitalcina* populations are allopatric and mostly separated by unsuitable habitat, it can be assumed that interspecific gene flow is minimal. As such, incongruence in gene tree topologies should largely reflect incomplete lineage sorting, consistent with the assumptions of most multispecies coalescent methods (Fujita et al. 2012). Conversely, extreme population genetic structuring across naturally fragmented habitats may represent a deviation from model assumptions (Niemiller et al. 2012, Hedin et al. 2015).

In this research we first use species discovery approaches to formulate alternative species delimitation hypotheses for the *Sitalcina sura* group. Based on genetic species delimitation results, supported by consideration of morphology, two new species and the previously unknown male of *Sitalcina borregoensis* are described. A time-calibrated multilocus species tree is used as a framework to interpret the biogeographic history of the *Sitalcina sura* group. We hypothesize that this history is linked to both plate tectonics and southern ancestry, similar to elements of the MTG.

## Methods

### Taxon sampling

Fieldwork was conducted in the winter and spring months when surface microhabitats were most suitable for successful collections. Voucher specimens used for morphological study and species descriptions were preserved in 80% ETOH, while those used in genetic analyses were preserved in 100% ETOH at -80 °C. All described species of the *Sitalcina sura* group were collected from at or near type localities (Ubick and Briggs 2008). This sampling included previously discussed (Ubick and Briggs 2008), but undescribed, specimens from the Santa Ynez Mountains, California. In addition, several new populations from California and Arizona were discovered in appropriate habitats. Two specimens per locality were used for phylogenetic analyses when available, with the final sample including 29 ingroup specimens from 16 localities (Figure 1, Suppl. material 1: Table S1). Locality data for all specimens are also available on the Symbiota Collections of Arthropods Network (http://symbiota4.acis.ufl.edu/scan/portal/index.php). Specimens are housed in the San Diego State Terrestrial Arthropod Collection (with SDSU_TAC or SDSU_OP catalog numbers); type specimens are deposited at the California Academy of Sciences (CAS). *Sitalcina californica* (Banks, 1893) and *Sitalcina lobata* Goodnight & Goodnight, 1942 were used as outgroup taxa in all analyses. Here we accept the hypothesis that *Sitalcina* is monophyletic, as argued on morphological grounds by Ubick and Briggs (2008).

### DNA isolation, amplification, and sequencing

Genomic DNA was extracted from leg tissue (2–3 legs) using the Qiagen DNeasy Kit (Qiagen, Valencia, CA). DNA fragments for mitochondrial cytochrome oxidase I (COI) and nuclear 28S rRNA, plus five additional protein-coding nuclear genes, were amplified using PCR (Table 1). Primers and cycling conditions are reported in Suppl. material 1: Table S2. Primers for protein-coding nuclear genes targeted terminal exon plus associated 3’ untranslated regions (3’-UTRs), and were developed by comparing transcriptomes of *Sitalcina lobata* (Hedin et al. 2012) to the phalangodid taxon *Texella bifurcata* (Briggs, 1968) (SRA numbers reported in Emata and Hedin 2016). Both transcriptomes were generated using Illumina short-read technology, and assembled using Trinity software (Grabherr et al. 2011). PCR products were purified using Millipore plates, Sanger sequenced in both directions at Macrogen USA (Rockville, MD), and edited using Geneious Pro 6 (Kearse et al. 2012). Sequences were aligned using MUSCLE (Edgar 2004), with the exception of 28S data, which were aligned using MAFFT (Katoh and Standley 2013) and the G_INS-I alignment algorithm (Wilm 2006). Alleles from heterozygous nuclear sequences were inferred using PHASE 2.1.1 (Stephens et al. 2001, Stephens and Scheet 2005), with each analysis repeated twice to ensure consistent results.

**Table 1.** Gene name, matrix completeness, aligned length, parsimony informative sites (ingroup), evolutionary and clock models.

Gene name	*Ixodes* homolog	Matrix % Complete	Aligned length	PI sites	Model of evolution	Clock model
Ecotropic viral integration site protein, putative	ISCW 021220	75%	508 bp	58	HKY+I	Relaxed
Protein phosphatase 2A regulatory subunit A, putative	ISCW 003443	>75%	242 bp	37	HKY+ Γ	Strict
RING finger protein, putative	ISCW 003817	>95%	214 bp	32	GTR+I	Strict
Protein transport protein Sec24A, putative	ISCW 016134	>95%	364 bp	37	GTR+ Γ	Relaxed
Neuromusculin, putative	ISCW 006547	>95%	197 bp	22	HKY+ Γ	Relaxed
28S		>95%	1094 bp	71	GTR+I+ Γ	Relaxed
COI (all)		100%	549 bp	249	GTR+ Γ	Relaxed
COI (pos1)					GTR+ Γ	
COI (pos2)					HKY+I+ Γ	
COI (pos 3)					GTR+Γ	

**Note**: PI, parsimony informative.

### Gene trees and genetic clustering

Models of DNA sequence evolution were chosen using jModeltest2 (Guindon and Gascuel 2003, Darriba et al. 2012), with the Akaike Information Criterion (AIC) used to select models (Table 1). Mitochondrial COI gene tree analyses were conducted using both codon partitioned and un-partitioned models. Gene trees for individual loci were reconstructed using MrBayes 3.2 (Ronquist et al. 2011) run on the Cyber Infrastructure for Phylogenetic Research (CIPRES; Miller et al. 2010). Bayesian MCMC analyses for each gene were run for 50 million generations sampling every 5000 generations. Each analysis was repeated twice to confirm results. ESS values along with –ln*L* scores were evaluated for convergence using Tracer v.1.6 (Rambaut et al. 2014). A 50% majority rule consensus tree from the resulting posterior distribution was constructed for each gene region.

We used genetic clustering in the initial “discovery” phase of species delimitation, a procedure common in the recent literature (reviewed in Carstens et al. 2013). STRUCTURE 2.3.4 (Pritchard et al. 2000) was used to analyze biallelic nuclear genotypic data. Each unique haplotype was treated as a single allele and was called using SNAP-map (Aylor et al. 2006). STRUCTURE runs were conducted with 20 iterations for multiple K values (K = number of genetic clusters). Separate runs were conducted with coastal clade species (see Results) with a K of 1-9, and desert clade species (see Results) with a K of 1-8. Analyses were run for 100,000 generations, with the first 10,000 generations removed as burnin. Analyses were run using both a no-admixture model (assumes each individual comes from one of the K distinct populations) and an admixture model (allows for population admixture). All other priors were left as default. Structure Harvester (Earl 2012) was used to find the best-fit K utilizing the ∆K method of Evanno et al. (2005). Data were summarized using the *FullSearch* algorithm of CLUMPP (Jakobsson and Rosenberg 2007), and visualized with DISTRUCT (Rosenberg 2004).

### Species trees and divergence times

Multilocus species trees were reconstructed using *BEAST 1.8 (Drummond et al. 2012). *A priori* species limits were based on genetic clusters identified by STRUCTURE admixture and no-admixture model results. For each analysis, substitution models, clock models, and trees were unlinked across loci. A best-fit model of molecular evolution was applied to each gene region (Table 1), with mitochondrial COI partitioned by codon and a Yule process applied for the species tree prior. Clock rate parameters were examined using Tracer v1.6 (Rambaut et al. 2014). If the 95% highest posterior density (HPD) of the coefficient of variation for any individual gene included zero (indicating that a strict molecular clock could not be statistically rejected), a strict clock was used for that gene region. Subsequent analyses were run with the appropriate uncorrelated relaxed or strict clock models (Table 1). Analyses were run for 200 million generations logging every 2000 generations. ESS values along with –ln*L* scores were evaluated for convergence using Tracer. Each species tree analysis was repeated twice to ensure consistent results. Posterior distributions of repeated runs were combined using LogCombiner, and a maximum clade credibility (MCC) tree was constructed from the resulting combined posterior distributions using TreeAnnotator.

Divergence time analyses were performed in *BEAST (Drummond et al. 2012). Because of general uncertainty in rates of molecular evolution in Laniatorean harvestmen, we conducted three separate analyses to provide bounds on possible dates. First, a well-accepted arthropod COI clock rate of 3.54% per million years (Ma) (arithmetic mean of branch rates (ucld) setting in *BEAST = 0.0178) was specified for the partitioned COI data (Papadopoulou et al. 2010). Second, the unpartitioned COI rate of Papadopoulou et al. (2010) was used (ucld mean = 0.0169). Finally, a slower COI rate calculated for the laniatorean genus *Sclerobunus* (ucld mean = 0.01115) was specified for the COI data partition (Derkarabetian et al. 2010). This latter rate was inferred indirectly based on combined biogeographic and fossil calibrations. For each analysis, a Yule process was specified for the species tree prior and a pairwise linear and constant root was applied for the population model, with a uniform distribution on the upper and lower bounds of the age. Substitution models, clock models, and trees were unlinked across loci. The appropriate model of molecular evolution and clock-like rate was applied to each gene fragment (see above). Analyses were run for 50 million generations sampling every 500, and repeated to confirm consistent results. Repeated runs were combined using LogCombiner, and a MCC tree was constructed using TreeAnnotator.

### Genetic species delimitation


 Bayes factor delimitation (BFD, Grummer et al. 2014) allows testing of alternative species delimitation models by assigning individuals to different lineages, with subsequent comparisons of marginal likelihoods of alternative models. We tested the alternative species delimitation models summarized in Table 2 – these alternative models are based on a combination of STRUCTURE results, geographic considerations, and prior taxonomy. Marginal likelihood estimations were run for 100,000 generations, sampled every 1000 with 100 steps using path sampling (Lartillot and Philippe 2006) and stepping stone (Xie et al. 2011) methods. A comparison of marginal likelihoods was conducted using Bayes factors, with values above 10 considered as decisive support (Kass and Raftery 1995).

**Table 2.** Alternative species delimitation hypotheses used in BFD analyses.

Hypothesis	Distinct Species (total in parentheses)	Motivation
H1	Glen Oaks, Santa Ynez, *Sitalcina borregoensis*, Borrego Palm Canyon, *Sitalcina catalina*, *Sitalcina chalona*, *Sitalcina flava* Topanga, *Sitalcina flava* Piuma + Palos Verdes, *Sitalcina peacheyi*, *Sitalcina peacheyi* MAD, *Sitalcina rothi*, *Sitalcina sura*, *Sitalcina seca* (13 species)	Following STRUCTURE admixture model
H2	Glen Oaks, Santa Ynez, *Sitalcina borregoensis*, Borrego Palm Canyon, *Sitalcina catalina*, *Sitalcina chalona*, *Sitalcina flava* Topanga, *Sitalcina flava* Piuma + Palos Verdes, *Sitalcina peacheyi*, *Sitalcina peacheyi* MAD, *Sitalcina rothi*, ***Sitalcina sura*** + ***Sitalcina seca*** (12 species)	Adjacent *Sitalcina sura* + *Sitalcina seca* considered a single species
H3	Glen Oaks, Santa Ynez, ***Sitalcina borregoensis*** + Borrego Palm Canyon, *Sitalcina catalina*, *Sitalcina chalona*, *Sitalcina flava* Topanga, *Sitalcina flava* Piuma + Palos Verdes, *Sitalcina peacheyi*, *Sitalcina peacheyi* MAD, *Sitalcina rothi*, *Sitalcina sura*, *Sitalcina seca* (12 species)	Anza-Borrego specimens considered a single species
H4	Glen Oaks, Santa Ynez, *Sitalcina borregoensis*, Borrego Palm Canyon, *Sitalcina catalina*, *Sitalcina chalona*, *Sitalcina flava* Topanga, *Sitalcina flava* Piuma + Palos Verdes, *Sitalcina peacheyi* + *Sitalcina peacheyi* MAD, *Sitalcina rothi*, *Sitalcina sura*, *Sitalcina seca* (12 species)	*Sitalcina peacheyi* as single species
H5	Santa Ynez, *Sitalcina borregoensis*, Borrego Palm Canyon, *Sitalcina catalina*, *Sitalcina chalona*, *Sitalcina flava* Topanga, *Sitalcina flava* Piuma + Palos Verdes, *Sitalcina peacheyi*, *Sitalcina peacheyi* MAD, Glen Oaks + ***Sitalcina rothi***, *Sitalcina sura*, *Sitalcina seca* (12 species)	Coastal STRUCTURE results + Desert no-admixture model results
H6	Glen Oaks, Santa Ynez, *Sitalcina borregoensis*, Borrego Palm Canyon, *Sitalcina catalina*, *Sitalcina chalona*, Palos Verdes + ***Sitalcina flava***, *Sitalcina peacheyi*, *Sitalcina peacheyi* MAD, *Sitalcina rothi*, *Sitalcina sura*, *Sitalcina seca* (12 species)	*Sitalcina flava* and Palos Verdes as single species
H7	Glen Oaks, *Sitalcina borregoensis*, Borrego Palm Canyon, *Sitalcina catalina*, *Sitalcina chalona*, Santa Ynez + *Sitalcina flava* + Palos Verdes, *Sitalcina peacheyi*, *Sitalcina peacheyi* MAD, *Sitalcina rothi*, *Sitalcina sura*, *Sitalcina seca* (11 species)	coastal southern CA a single species
H8	Glen Oaks, Santa Ynez, ***Sitalcina borregoensis*** + Borrego Palm Canyon, *Sitalcina catalina*, *Sitalcina chalona*, *Sitalcina flava* Topanga, *Sitalcina flava* Piuma + Palos Verdes, *Sitalcina peacheyi* + *Sitalcina peacheyi* MAD, *Sitalcina rothi*, *Sitalcina sura*, *Sitalcina seca* (11 species)	*Sitalcina peacheyi* as single species; Anza-Borrego specimens considered a single species
H9	*Sitalcina borregoensis* + Borrego Palm Canyon, *Sitalcina catalina*, *Sitalcina chalona*, *Sitalcina flava* +Santa Ynez + Palos Verdes, *Sitalcina peacheyi* + *Sitalcina peacheyi* MAD, *Sitalcina rothi* + Glen Oaks, *Sitalcina sura* + *Sitalcina seca* (7 species)	Putative species grouped with geographic neighbors

### Niche modeling

With incomplete knowledge of the full distribution of the *Sitalcina sura* group, possible regions with additional new populations or species were identified via ecological niche modeling (ENM). DIVA-GIS (Hijmans et al. 2004) and Maxent (Phillips and Dukid 2008) were used to construct niche models, using known localities for the species group as input. Niche models have previously successfully predicted the potential habitats of animal taxa with small and fragmented distributions (e.g., Rissler and Apodaca 2007, Bond and Stockman 2008, Bond 2012). Predicted distributions of the *Sitalcina sura* group were reconstructed using altitudinal and climatic layers for nineteen quarterly and annual measurements of temperature and precipitation (Bioclimatic layers 1-19), obtained from the WorldClim dataset (Hijmans et al. 2005). A jackknife analysis was conducted in Maxent to discover the most likely environmental factors impacting species’ distributions. Output from Maxent was converted to raster format and reclassified to binary presence/absence in ArcMap 10.3 (ESRI) using the 10 percentile training presence logistic threshold.

### Study of morphology

Male penises that were not protruding from the genital operculum were physically extracted using a blunt insect micro pin. Exposed penises were placed in room temperature (or hot) 10% KOH for 1-2 minutes for expansion. Female ovipositors were exposed using the same blunt pin procedure. Specimens were imaged using a Quanta 450 scanning electron microscope (SEM) after being mounted and coated with 20nm platinum. One or two specimens were used for SEM as needed. Whole specimen digital images were captured using a Visionary Digital BK plus system (http://www.visionarydigital.com). Individual images were merged into a composite image using Helicon Focus 6.2.2 software (http://www.heliconsoft.com/heliconfocus.html). Specimen measurements were taken with an SZX12 Olympus dissecting scope equipped with an ocular micrometer, at 50× magnification.

## Results

### Data characteristics

DNA sequence data were collected for two outgroup taxa (data for *Sitalcina lobata* from transcriptomes), eight described species from the *Sitalcina sura* group, and four novel geographic populations with morphological features placing them in the *Sitalcina sura* group (see diagnostic features below). Not all specimens were successfully amplified for all gene regions, resulting in some missing data (Table 1). All heterozygous nuclear sequences were PHASED with 100% certainty. GenBank accession numbers for unphased sequences are reported in Suppl. material 1: Table S3, and phased data matrices have been submitted to the Dryad Digital Repository (http://dx.doi.org/10.5061/dryad.4gk4f).

### Gene trees and genetic clustering

**Figure 3.**
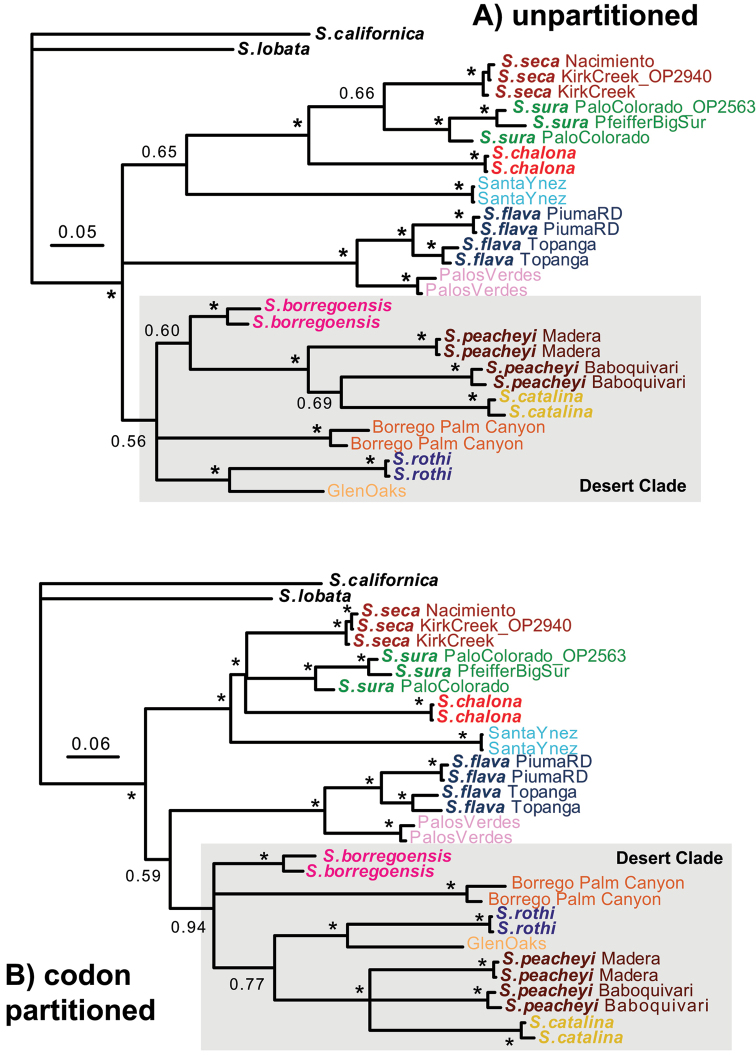
Mitochondrial COI gene trees. Results based on **A** un-partitioned and **B** codon partitioned analyses. Asterisks indicate posterior probabilities above 0.95. Members of desert clade (see text) highlighted.

Mitochondrial COI gene trees were reconstructed using both a codon partitioned and un-partitioned model, and are generally topologically similar (Figure 3). A clade that includes southern California desert populations together with Arizona sky-island populations (hereafter named the “desert” clade) is recovered in both analyses. Geographically proximate populations from canyons in the Anza Borrego desert (Borrego Palm Canyon, *Sitalcina borregoensis*) are surprisingly genetically divergent, with average Kimura 2-parameter distances exceeding 17% (calculated using MEGA6, Tamura et al. 2013), and do not form a clade on COI trees. An important incongruence between codon partitioned versus un-partitioned results concerns the uncertain placement of the *Sitalcina flava* + Palos Verdes clade. These populations are consistently placed with other coastal CA populations (hereafter named the “coastal” clade, see below) in nuclear gene tree analyses, but group with the desert clade with low support (posterior probability (PP) = 0.59) in codon partitioned COI analyses.

**Figure 4.**
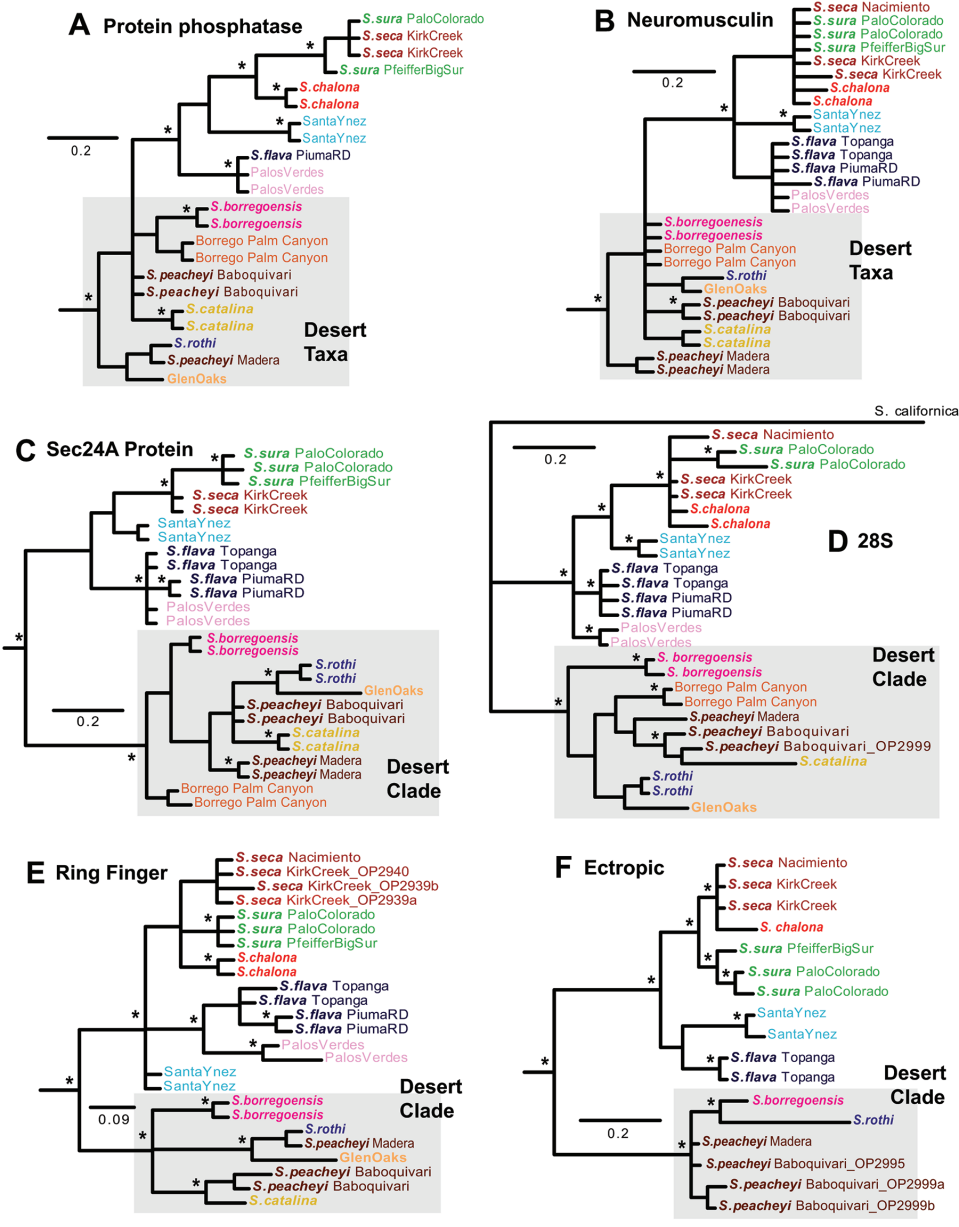
Nuclear gene trees. Individual genes include **A** Protein phosphatase 2A regulatory subunit A **B** Neuromusculin **C** Protein transport protein Sec24A **D** 28S, **E** RING finger protein, and **F** Ecotropic viral integration site protein. Outgroups trimmed from all trees except for 28S. Asterisks indicate posterior probabilities above 0.95. Desert taxa shaded.

Despite expected variance in nuclear gene tree topologies, several general trends are apparent. First, a coastal CA clade is recovered (generally with strong support, PP > 0.95) in all six nuclear gene trees (Figure 4). This clade includes *Sitalcina sura*, *Sitalcina seca*, *Sitalcina chalona*, *Sitalcina flava*, Santa Ynez, and Palos Verdes populations. Specimens from Santa Ynez are genetically distinct in all nuclear gene trees. A desert clade is strongly supported in four of six nuclear gene trees; paraphyly of this group in two gene trees may reflect a gene tree rooting issue. Within the desert clade, Borrego Palm Canyon specimens are genetically distinct from neighboring *Sitalcina borregoensis* in four gene trees where both populations were sampled (Figure 4).

**Figure 5.**
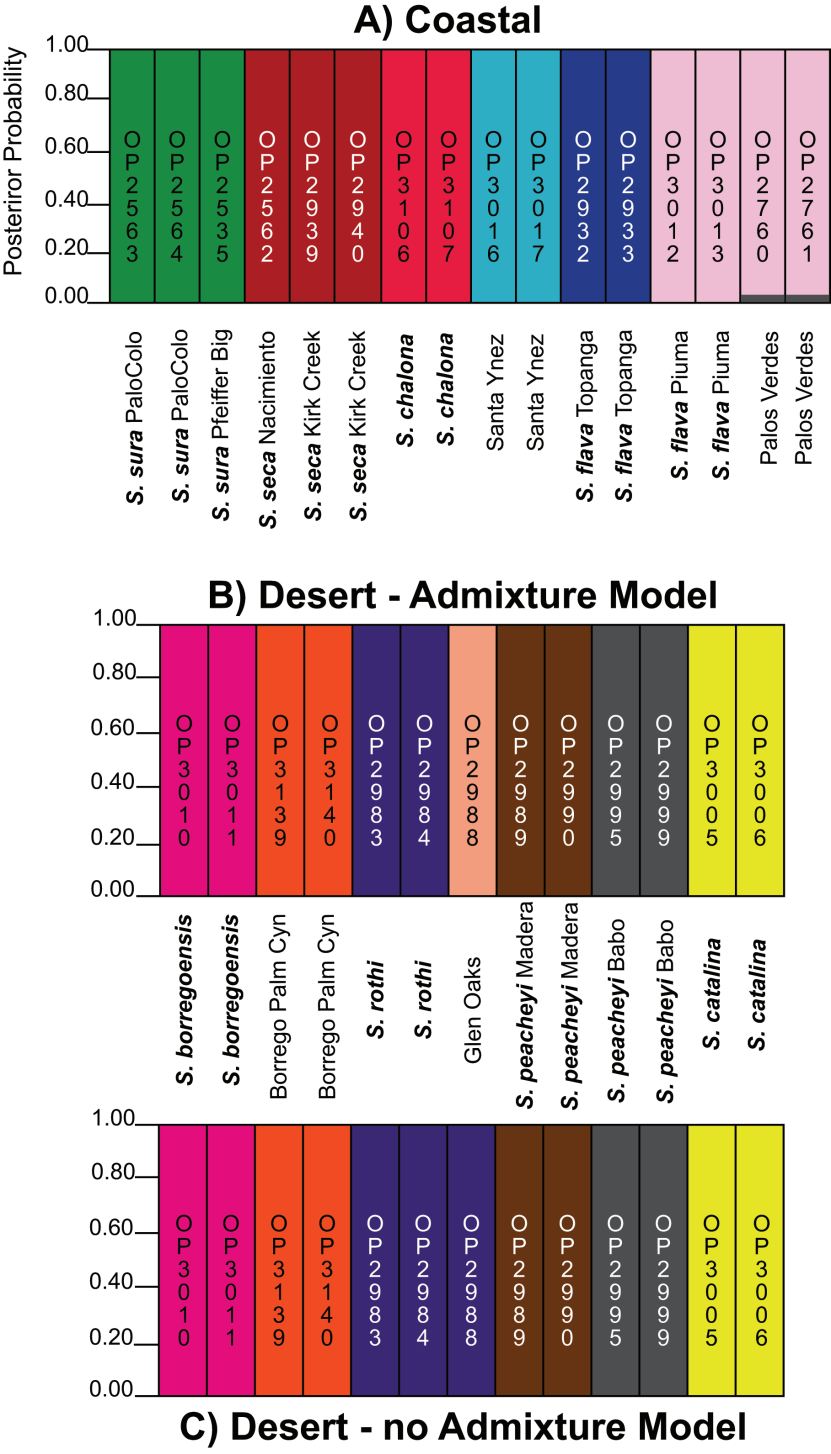
STRUCTURE results. Results for **A** coastal clade using admixture and no admixture models (K=6) **B** desert clade using admixture model (K=7), and **C** desert clade using no admixture model (K=6).

STRUCTURE analyses for coastal clade specimens favor a K = 6 model (Figure 5A). Both no-admixture and admixture models place *Sitalcina sura*, *Sitalcina seca*, *Sitalcina chalona*, and Santa Ynez individuals into four separate genetic clusters. Specimens from Palos Verdes and *Sitalcina flava* from Piuma Road group together into a fifth genetic cluster, while Topanga Canyon *Sitalcina flava* specimens represent a sixth cluster. The STRUCTURE admixture model favors all discrete desert populations (either found on isolated mountain ranges or in isolated desert canyons) as unique genetic clusters (K = 7, Figure 5B). A no-admixture model favors K = 6, grouping geographically adjacent Glen Oaks and *Sitalcina rothi* specimens (Figure 5C).

### Species trees and divergence times

**Figure 6.**
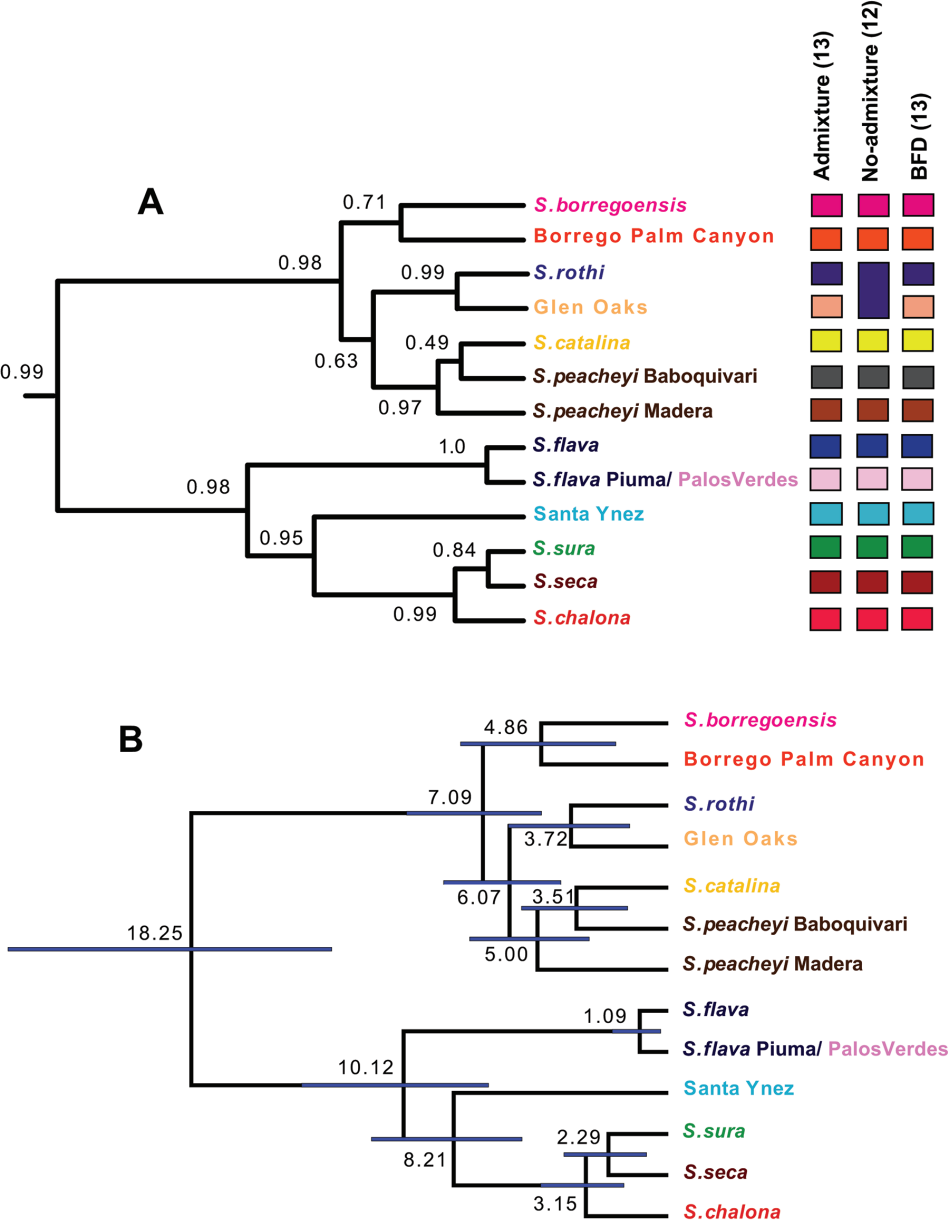
*BEAST species trees. **A** Includes summary of STRUCTURE and BFD species delimitation results. Outgroup taxa not shown **B** Includes divergence time estimates based on Papadopoulou et al. (2010) partitioned COI rate. Ages (in millions of years) and bars indicating 95% highest posterior density (HPD). Outgroup taxa not shown.

*BEAST analyses conducted with admixture STRUCTURE clusters as *a priori* species recover desert and coastal clades with strong support (PP > 0.95, Figure 6A). Relationships among coastal taxa are well supported, with an internal topology that mirrors geography (early-diverging southern lineages, Santa Ynez intermediate, derived northern lineages). Relationships between desert taxa are less strongly supported, with CA desert canyon populations forming a clade sister to montane Arizona populations.

Using the Papadopoulou et al. (2010) partitioned COI rate, the estimated divergence time for the most recent common ancestor (tMRCA) of the *Sitalcina sura* group is ~18 Ma, the tMRCA for the coastal clade is ~10 Ma, and the tMRCA for the desert clade is ~7 Ma (Figure 6B). The Papadopoulou et al. (2010) unpartitioned COI rate provides very similar time estimates, while the slower *Sclerobunus* rate results in clearly older time estimates for these same nodes (Table 3). In general, we have no *a priori* reason to favor one of these rates, given the general lack of knowledge of rates of molecular evolution in laniatorean harvestmen. However, we favor the younger dates for three reasons. First, the Papadopoulou et al. (2010) rates result in date estimates that coincide with important biogeographic events in the region (see Discussion). Second, various temporal analyses conducted by Emata and Hedin (2016) for the California phalangodid genus *Calicina* also showed that the Papadopoulou et al. (2010) rate provided biologically realistic dates for a California taxon, while slower rates suggested unrealistically old divergence dates. Finally, we note that recent studies of *Sclerobunus* using whole genome SNP data (Derkarabetian et al. 2016) provide younger divergence ages than previously hypothesized in that system, suggesting that the Derkarabetian et al. (2010) COI rate may have been underestimated. All of these arguments are *ad hoc*, with resolution in the *Sitalcina* system ultimately requiring additional data.

**Table 3.** Divergence time estimates from alternative COI molecular clock rates.

Model	Papadopoulou partitioned COI rate	Papadopoulou unpartitioned COI rate	Derkarabetian COI rate
tMRCA *Sitalcina sura* group	18.25 (12.88–25.26)	19.36 (13.58–26.47)	29.43 (19.56–40.87)
tMRCA coastal clade	10.12 (6.88–14.03)	10.98 (7.56–14.85)	16.25 (10.57–23.07)
tMRCA desert clade	7.09 (4.85–10.01)	7.49 (5.09–10.37)	11.39 (7.64–16.04)
tMRCA *Sitalcina borregoensis*, *Sitalcina oasiensis*	4.86 (2–7.95)	5.32 (2.09–8.57)	7.87 (3–13.22)

### Niche modeling

**Figure 7.**
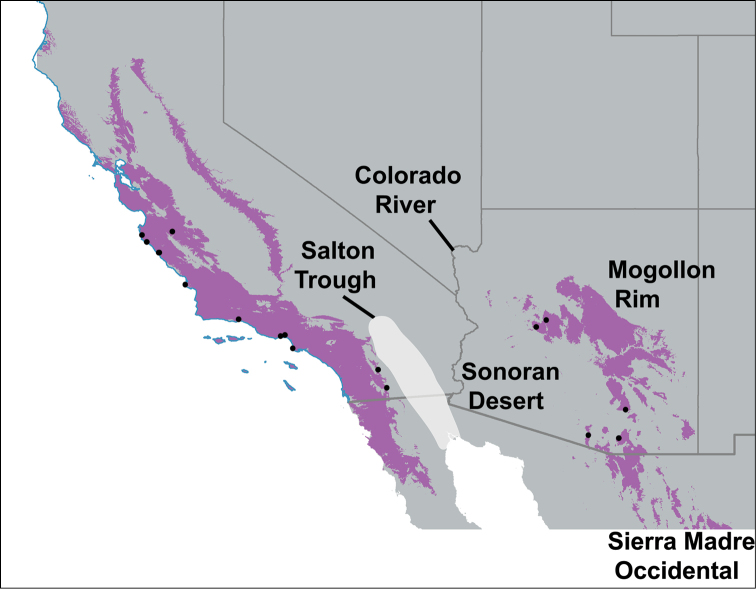
Binary ENM for *Sitalcina sura* species group. Purple regions indicate predicted habitat and dots indicate collection localities used in model estimation. Major geographic features highlighted.

Precipitation in the coldest quarter (BIO19) and precipitation in the driest month (BIO14) were the best predictors of *Sitalcina sura* group distributions. ENMs based on these variables provide a visualization of possible sampling gaps within the *Sitalcina sura* group (Figure 7). In California and Baja California Norte, areas of high suitability that represent possible gaps include the Santa Ynez Mountains northwest of Santa Barbara, and the southern Santa Lucia Mountains. Some of these habitats are occupied by *Sitalcina californica*, which has an apparently exclusive distribution with members of the *Sitalcina sura* group (i.e., no sympatry has ever been recorded, Figure 1). Similarly, we expect *Sitalcina lobata* to occupy the predicted suitable habitats of coastal southern CA and northern Baja (Figure 1). In these particular cases, we hypothesize that the ENM has over-predicted the distribution of the *Sitalcina sura* group. California desert canyon habitats, both north and south of our sampled populations, have predicted suitability. In Arizona and northern mainland Mexico (Sonora), unsampled populations are predicted to occur in montane habitats between known *Sitalcina catalina* - *Sitalcina rothi* populations, and south of our current sample. We are not aware of *Sitalcina* records from northern mainland Mexico, but based on habitat and distribution of other regional sky-island animal taxa (e.g., Maddison and McMahon 2000, Bryson et al. 2011, Bryson et al. 2013, Grummer et al. 2014), we expect *Sitalcina* to occur in the mountains of Sonora.

### Genetic species delimitation

BFD results support a 13 species model, following the K = 6 STRUCTURE model for coastal taxa and the K = 7 model for desert taxa (Tables 2 & 4, Figure 6). This 13 species model supports three new populations as putative species (Borrego Palm Canyon, Santa Ynez, Glen Oaks), and the division of both *Sitalcina peacheyi* and *Sitalcina flava* into separate species. This model is decisively supported over alternative species delimitation models (Table 4). In translating BFD results into formal taxonomy, we have taken a conservative approach, also considering support from other lines of evidence. Because Glen Oaks is known only from a single adult female specimen, we defer formal species description to a later date, when additional specimens can be collected and studied. Further attention should be also directed at the geographically disjunct Palos Verdes population, which is genetically distinct from *Sitalcina flava* for multiple genes (Figures 3, 4). Finally, because we failed to find obvious morphological differences between disjunct populations of *Sitalcina peacheyi* and *Sitalcina flava*, and because multispecies coalescent models might oversplit genetically structured populations (Niemiller et al. 2012, Hedin et al. 2015), we do not further split *Sitalcina peacheyi* and *Sitalcina flava* at this time.

**Table 4.** BFD results.

Model	Path Sampling	Bayes Factor PS	Stepping Stone	Bayes Factor SS
H1	-12137	**NA**	-11953	**NA**
H2	-12387	499	-12366	827
H3	-12308	342	-12252	598
H4	-12258	241	-12137	369
H5	-12303	331	-12235	563
H6	-12235	196	-12129	351
H7	-12264	253	-12140	373
H8	-12352	429	-12300	693
H9	-12287	300	-12131	357

**Note**: Hypotheses as in Table 2.

Genetically distinct and morphologically diagnosable populations from Borrego Palm Canyon and Santa Ynez are formally described below. Also, previously unknown males of *Sitalcina borregoensis* are described. Following Ubick and Briggs (2008), we emphasize both somatic and genitalic characters in our descriptions.

## Taxonomy

Morphological abbreviations (following Ubick and Briggs 2008): 1. Somatic morphology: AT = anterior tubercles of scute, BL = body length (viewed laterally), EM = eye mound, GO = genital operculum, Fm = femur, SL = scute length (from front edge of EM to posterior edge of scute, viewed dorsally), SW = scute width at widest point, viewed dorsally), TC = tarsal count, TrIV = trochanter of leg IV. 2. Penis morphology: DL = dorsal lobe of glans, PSL = parastylar lobe(s) of glans, S = stylus, VP = ventral plate of penis, VS = ventral setae of ventral plate, AS = apical spine of ventral plate. 3. Ovipositor morphology: OV = ovipositor, OVM = ovipositor microspines, OVS = ovipositor apical setae. All measurements are in millimeters unless noted otherwise.

### *Sitalcina* Banks, 1911

**Diagnosis.** As presented in Ubick and Briggs (2008).

### *Sitalcina sura* Group

**Diagnosis.** Members of the *Sitalcina sura* group are distinguished from related *Sitalcina californica* and *Sitalcina lobata* by the following characters (Ubick and Briggs 2008): Both sexes possess a short row of dorsomesal asetose tubercles on the palpal Fm. Males lack an AS, possess a bilobed PSL, and a curved to straight ectal spur on TrIV. Females with imbricate OVM (absent/weak in the (*Sitalcina chalona*, (*Sitalcina sura*, *Sitalcina seca*)) clade), and curved OVS with brush-like tips.

#### Sitalcina borregoensis

Taxon classificationAnimaliaOpilionesPhalangodidae

Briggs, 1968

**Figure 8.**
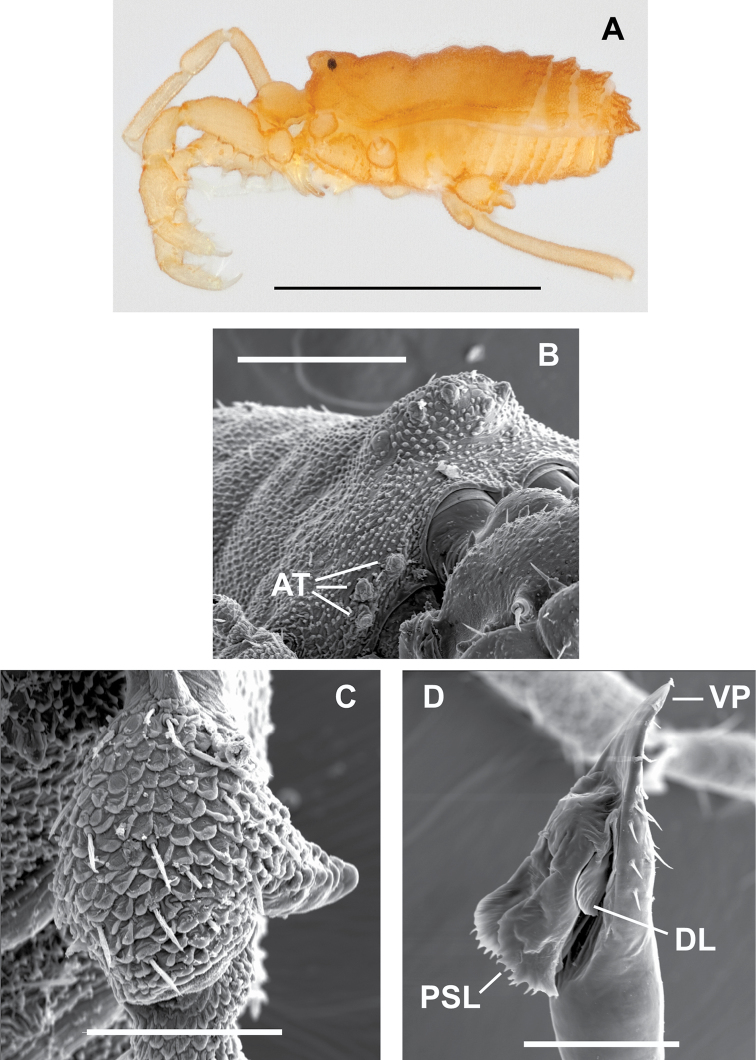
Male *Sitalcina borregoensis* (ETOH - SDSU_OP3011, SEM prep - SDSU_TAC000293) – **A** habitus **B** EM **C** TrIV **D** penis. Scale bar = 1 mm (**A**), 200 µm (**B**), 100 µm (**C, D**).

Figures: map Figure 1 ; habitus Figure 2B ; male Figure 8A–D

Sitalcina borregoensis Briggs, 1968: 30. Ubick and Briggs 2008: 22, fig. 25.

##### Type material examined.

**Holotype** female from California, San Diego County, Anza- Borrego Desert State Park, Mountain Palm Springs, collected by T. Briggs, April 5, 1967 (CAS).

##### Diagnosis.

This small-bodied species is most similar to *Sitalcina rothi* and *Sitalcina oasiensis*, with a low EM and a flattened body profile in both sexes. Females can be diagnosed by the moderately imbricate OVM. Males possess a TrIV spur that is approximately straight, and longer than in *Sitalcina oasiensis*. The distal end of PSL is conspicuously serrate.

##### Genetic data.

GenBank Accession numbers: KX064802, KX064803, KX064830, KX064831, KX064855, KX064856, KX064876, KX064898, KX064899, KX064924, KX064925, KX064954, KX064955.

##### Description.

FEMALE. As in Ubick and Briggs 2008.

MALE. Integument color pale orange, appendages lighter. Body finely rugose with a few large tubercles on posterior tergites, one pair anteriorly on EM; 3 pairs of AT. EM low, flattened, eyes present. Palpal Fm with median dorsobasal row of 4 asetose tubercles and one small mesal tubercle. Palpal megaspines: trochanter one ventral and small; Fm 3 ventrobasal, one mesodistal; patella 2 mesal, one ectal; tibia and tarsus 2 mesal, 2 ectal. TC 3-5-5-5.

Measurements taken from following specimens: SDSU_OP3011 (SDSU_OP3010): BL 1.32 (1.24). SL 0.75 (0.84), SW 0.75 (0.78). EM width 0.19 (0.18), height 0.12 (0.10). GO length 0.17 (0.14), width 0.17 (0.14). Leg II length - missing (2.78), Leg II/SL - missing (3.31). TrIV spur present, nearly straight. Penis VP entire, apically pointed, with 8 pairs of setae, AS absent; glans DL quadrate; PSL serrate distally; S not visible.

##### Other material examined.

New males were collected on 19 February, 2012 from the vicinity of the type locality. Suppl. material 1: Table S1 provides additional locality information for specimens examined.

##### Distribution and habitat.

Known only from the vicinity of Mountain Palm Springs, Anza Borrego Desert State Park. New collections are from a north-facing slope, under the first layer of granite rocks in a small ravine adjacent to a palm grove (Figure 2A).

#### Sitalcina oasiensis

Taxon classificationAnimaliaOpilionesPhalangodidae

DiDomenico & Hedin sp. n.

http://zoobank.org/12D14E94-D653-4340-A4AA-4D0ECD9E1400

**Figure 9.**
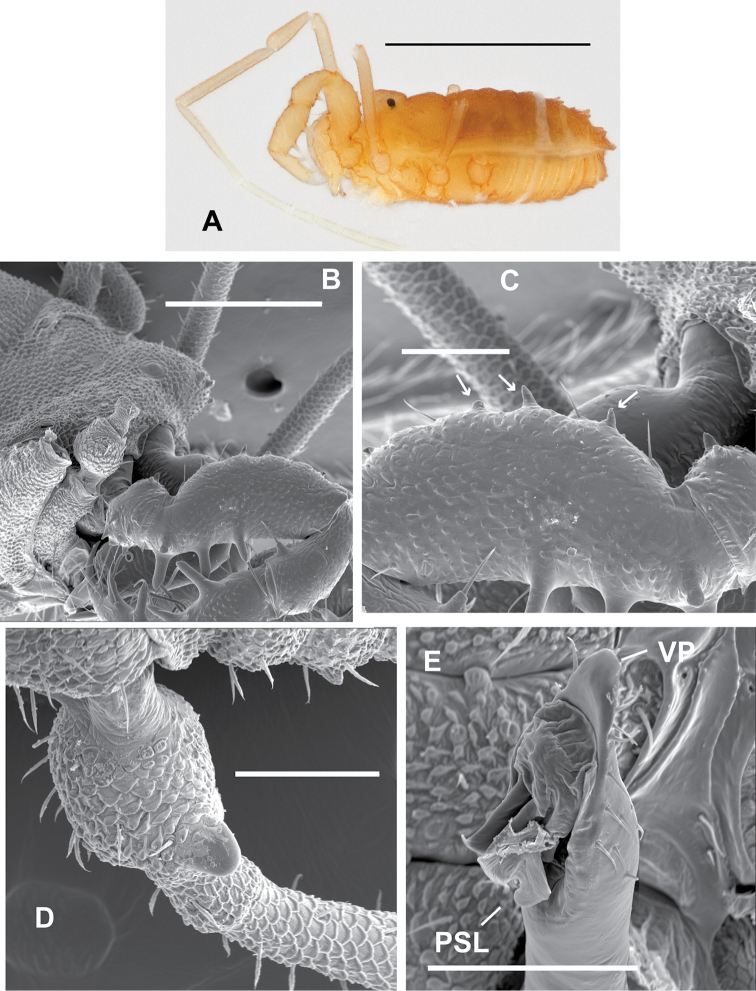
Male *Sitalcina oasiensis* (ETOH - SDSU_ OP3140, SEM prep - SDSU_TAC000289) – **A** habitus **B** EM **C** palpal Fm, asetose tubercles at arrows **D** TrIV **E** penis. Scale bar: 1 mm (**A**), 300 µm (**B**), 100 µm (**C–E**).

**Figure 10.**
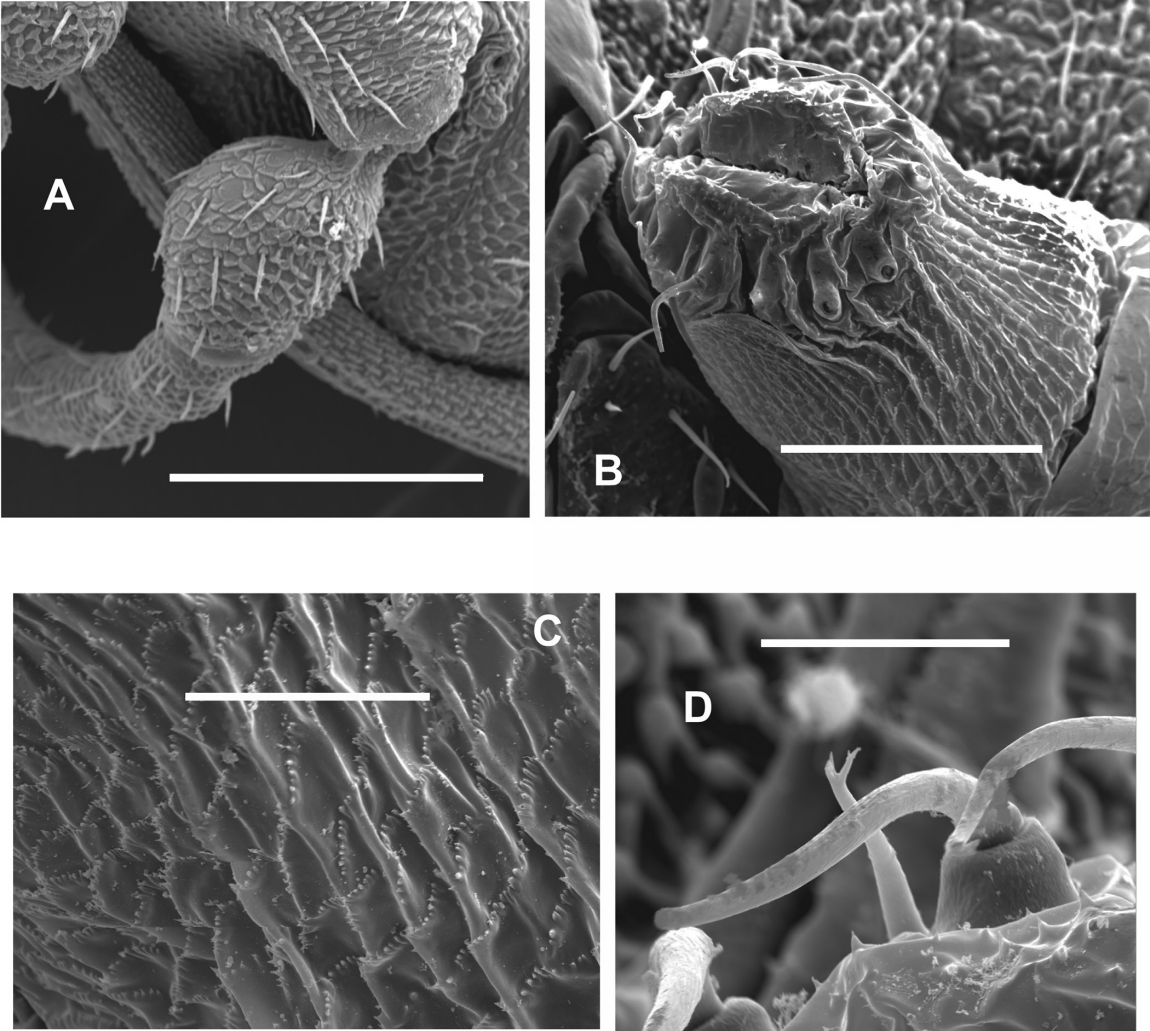
Female *Sitalcina oasiensis* (SDSU_TAC000290) – **A** TrIV **B** OV **C** OVM **D** OVS. Scale bar: 200 µm (**A**), 100 µm (**B**), 30 µm (**C**), 20 µm (**D**).

Figures: map Figure 1 ; male Figure 9A–E ; female Figure 10A–D

##### Type material.

**Holotype** male (SDSU_TAC000211, CASENT 9029998) from California, San Diego County, Anza-Borrego Desert State Park, Borrego Palm Canyon. N33.28025°, W116.43369° elev. ca. 430 m. Collected by A. DiDomenico, D. Carlson, S. Derkarabetian, S. Bejarano, February 23, 2013.

##### Etymology.

Named for the well-known palm oases of Borrego Palm Canyon.

##### Diagnosis.

Both sexes are small-bodied, with a low EM and a flattened body profile, similar to *Sitalcina borregoensis*. Females can be distinguished by the more strongly imbricate OVM. The male TrIV spur is approximately straight, shorter than in *Sitalcina borregoensis*.

##### Genetic data.

GenBank Accession numbers: KX064804, KX064805, KX064832, KX064833, KX064857, KX064858, KX064900, KX064901, KX064926, KX064927.

##### Description.

Integument color pale orange with lighter appendages. Body finely rugose with larger tubercles along tergal margins and small tubercles anteriorly on EM; 2-3 pairs of AT. EM low and rounded, eyes present. Palpal Fm with median dorsobasal row of 3 asetose tubercles and one small mesal tubercle. Palpal megaspines: trochanter 2 ventral; Fm 3 ventrobasal, one mesodistal; patella 2 mesal, one ectal; tibia and tarsus 2 mesal, 2 ectal. TC 3-5-5-5.

MALE. Holotype (paratypes SDSU_TAC000297, SDSU_TAC000298): BL 1.3 (1.13–1.28). SL 0.81 (0.75- 0.8), SW 0.78 (0.73–0.88). EM width 0.19 (0.20), height 0.11 (0.10- 0.13). GO length 0.14, width 0.15. Leg II length 2.7 (2.34–2.64), Leg II/SL 3.33 (2.93–3.12). TrIV spur short, straight. Penis VP entire, apically rounded, with 9 pairs of setae, AS absent; glans DL quadrate; PSL simple and bilobed, rounded at apical end; S not visible.

FEMALE paratype SDSU_TAC000299: BL 1.5. SL 0.8, SW 0.9. EM width 0.25, height 0.13. Leg II length 3.0, Leg II/SL 3.75. Strongly imbricate OVM, apical teeth absent, 7 pairs of OVS, curved, multifurcate.

##### Other material examined.

See Suppl. material 1: Table S1 for locality information for specimens examined.

##### Distribution and habitat.

Known only from the type locality. Specimens were collected from sparse desert chaparral habitat, under small rocks amongst larger granite boulders, NE-facing slope above palm oases.

#### Sitalcina ubicki

Taxon classificationAnimaliaOpilionesPhalangodidae

DiDomenico & Hedin sp. n.

http://zoobank.org/ECA3E4F6-7182-4974-8875-5FFF30EDB6B6

**Figure 11.**
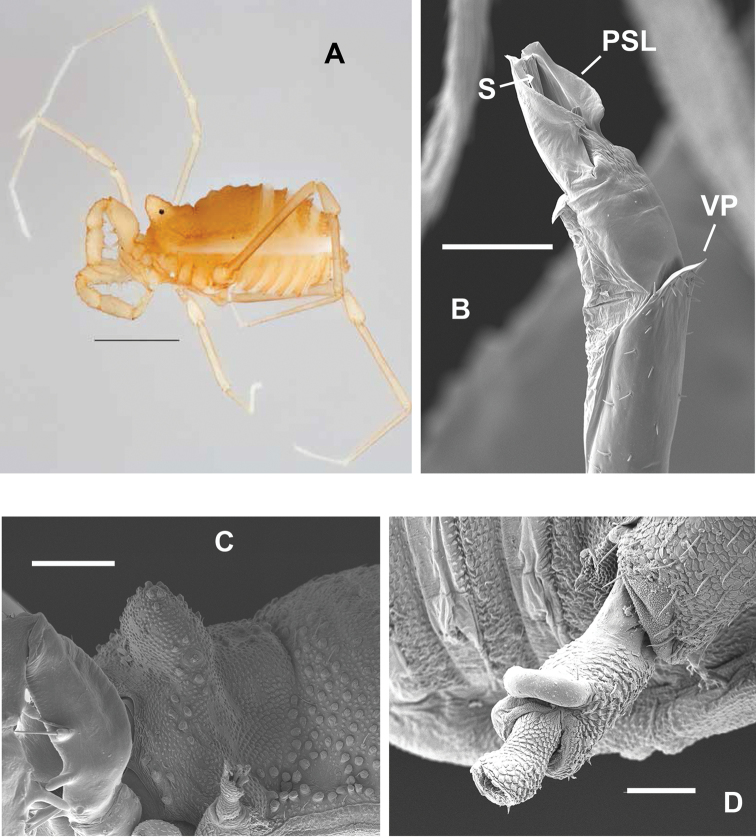
Male *Sitalcina ubicki* (ETOH - SDSU_TAC000216, SEM prep - SDSU_TAC000291) – **A** habitus **B** penis, stylus at arrow **C** EM **D** TrIV. Scale bar: 1 mm (**A**), 200 µm (**C**), 100 µm (**B, D**).

**Figure 12.**
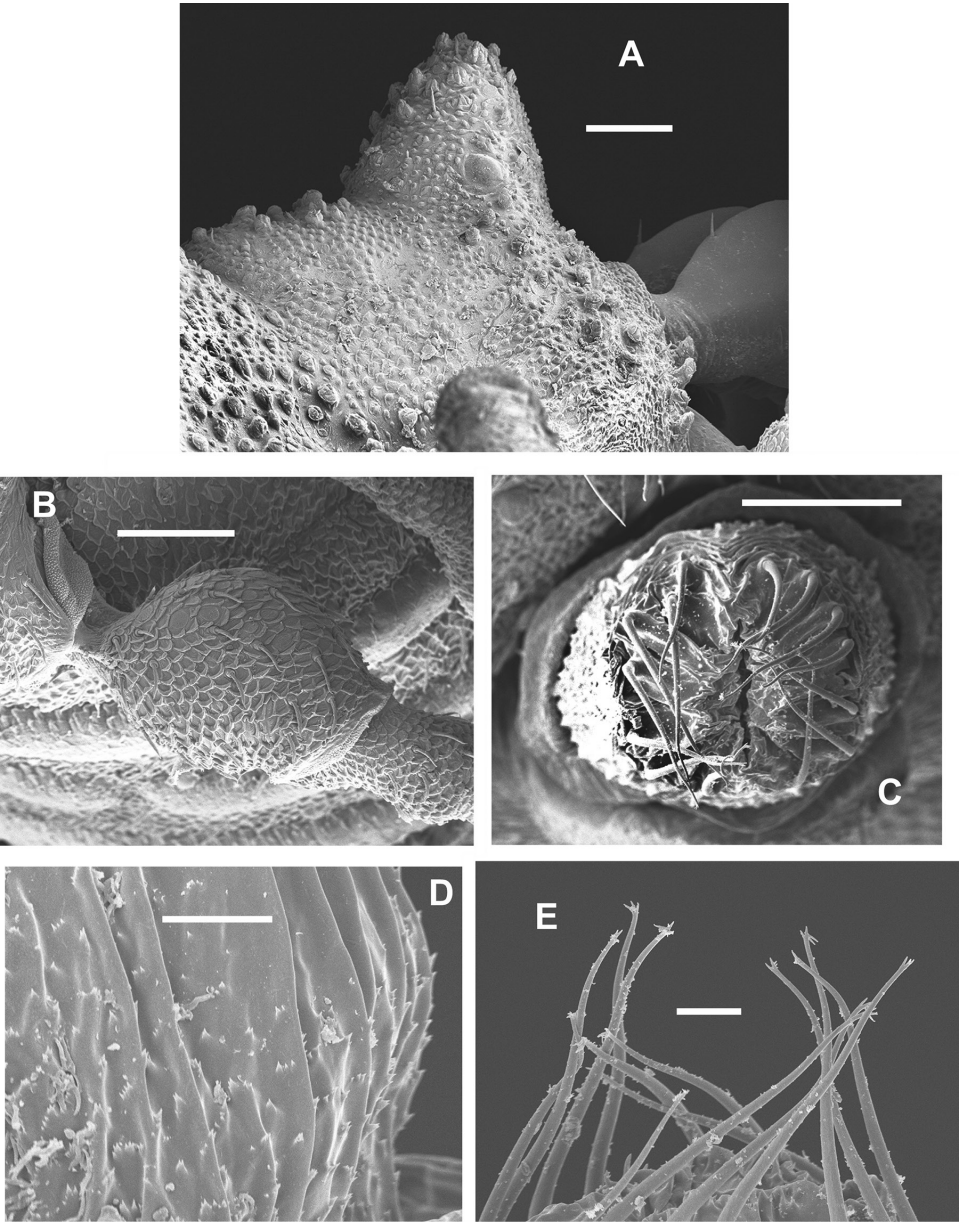
Female *Sitalcina ubicki* (SDSU_TAC000292) – **A** EM **B** TrIV **C** OV **D** OVM **E** OVS. Scale bar: 100 µm (**A, B, C**), 20 µm (**D, E**).

Figures: map Figure 1 ; male Figure 11A–D ; female Figure 12A–D

##### Type material.

**Holotype** male (SDSU_TAC000216, CASENT 9029999) from California, Santa Barbara County, Santa Ynez Mtns, Montecito, E Mountain Drive, 0.4 mi NE from jct. with Cold Spring Road N34.45496°, W119.65288°, elev. ca. 230 m. Collected by A. DiDomenico, K. Emata, E. Garcia, A. Schönhofer, February 18, 2012.

##### Etymology.

This species is named in honor of Darrell Ubick (CAS) whose foundational taxonomic research with *Sitalcina* made this project possible.

##### Diagnosis.

Similar to other members of the northern coastal clade (*Sitalcina sura*, *Sitalcina seca*, *Sitalcina chalona*) in body form, relatively large-bodied, with a tall and pointed, tuberculate EM. Male TrIV spur curved, penis PSL similar to but distinguishable from *Sitalcina chalona*.

##### Genetic data.

GenBank Accession numbers: KX064794, KX064795, KX064822, KX064823, KX064850, KX064851, KX064873, KX064890, KX064891, KX064916, KX064917, KX064946, KX064947

##### Description.

Integument color light orange with lighter appendages. Body finely rugose with larger tubercles along tergal margins and anteriorly on EM; 3-4 pairs of AT. EM tall, slightly pointed, eyes present. Palpal Fm with median dorsobasal row of 4 asetose tubercles and one small mesal tubercle. Palpal megaspines: trochanter 2 ventral; Fm 3 ventrobasal, one mesodistal; patella 2 mesal, one ectal; tibia and tarsus 2 mesal, 2 ectal. TC 3-5-5-5.

MALE. Holotype (paratypes SDSU_TAC000300, SDSU_TAC000301, SDSU_TAC000302): BL 2.1 (1.68–2.4). SL 1.14 (1.15–1.4), SW 1.3 (1.2–1.4). EM width 0.33 (0.35–0.4), height 0.23 (0.25–0.35). GO length 0.21, width 0.21. Leg II length 5.16 (4.2–5.12), Leg II/Scute Length 4.52 (3.14–3.66). TrIV spur short, curved. Penis VP entire, apically pointed, with 12 pairs of setae, AS absent; glans DL quadrate; PSL simple, rounded at apical end; S thick.

FEMALE. Paratypes SDSU_TAC000207 (SDSU_TAC000303, SDSU_TAC000304): BL 1.88 (1.7–2.0). SL 1.36 (1.0–1.25), SW 1.4 (1.25–1.35). EM width 0.36 (0.35), height 0.36 (0.2–0.25). Leg II length 4.48 (3.75–4.4), Leg II/Scute Length 3.29 (3.0–3.52). Slightly imbricate OVM, 6 pairs of OVS, straight, multifurcate.

##### Other material examined.

See Suppl. material 1: Table S1 for locality information of all specimens examined.

##### Distribution and habitat.

Known only from the vicinity of Montecito, Santa Ynez Mountains, Santa Barbara County. Specimens were found under stones, in narrow ravine with stream, in a *Quercus* and *Platanus* forest.

##### Note.

This population was mentioned by Ubick and Briggs (2008), but specimens were damaged and left undescribed. The new material collected for this study is from the same general vicinity as specimens studied by Ubick and Briggs (2008); northern edge of Montecito, off East Mountain Drive.

## Discussion

### Sitalcina remains poorly known

Species limits in the *Sitalcina sura* group were first studied by Ubick and Briggs (2008) based on consideration of somatic and genital morphology. In the current study, these and other species hypotheses (Table 2) were formally tested using multilocus DNA sequence data and a Bayes Factor Delimitation approach. With two newly described species, *Sitalcina* now includes a dozen species, and we expect future research to uncover additional undescribed taxa. Ultimately, we predict that *Sitalcina* diversity might approach that observed in the phalangodid genus *Calicina*, a taxon that includes over 25 short-range endemic species from central and northern California (Ubick and Briggs 1989, Emata and Hedin 2016).

Our results imply that some described species (e.g., *Sitalcina peacheyi*, *Sitalcina flava*) may actually comprise multiple cryptic species. This pattern is expected in a morphologically conservative group that occupies naturally fragmented habitats. This prediction would also apply to the geographically widespread *Sitalcina californica* (see Ubick and Briggs 2008), which has an interesting disjunct distribution in California (Figure 1). We have also discovered new geographic populations (e.g., Glen Oaks, Palos Verdes) that may represent new species. For both new populations and potential cryptic species, future research implementing genomic-scale datasets would be informative. Finally, our results confirm highly localized geographic speciation in these sedentary animals. Examples include geographically adjacent *Sitalcina seca*, *Sitalcina sura*, and *Sitalcina chalona*, as well as *Sitalcina borregoensis* plus *Sitalcina oasiensis*. If we extrapolate this pattern of localized divergence to regions where *Sitalcina* is likely, but currently unknown, this suggests many additional species. If *Sitalcina* is distributed like “beads on a string”, we are missing many of the beads.

*Sitalcina* specimens are sometimes very challenging to collect. Specimens only occur near the surface (i.e., under accessible rocks or woody debris) at certain times of the year, apparently migrating deep into the soil matrix as surface conditions dry (Ubick and Briggs 2008). This means that collections must take place in “good” years (e.g., non-drought years), in appropriate months, by experienced collectors in suitable microhabitats. An example is the Mountain Palm Springs population of *Sitalcina borregoensis*, which was first collected in 1967 (Ubick and Briggs 2008), but not recollected until 2012. Glen Oaks is another example, with only a single specimen collected despite over 3 hours of search time by a team of six experienced collectors. In this context, new populations of *Sitalcina* in poorly collected regions are likely, and we highlight the montane sky islands of northern Mexico and the desert canyons of southern California plus Baja California Norte as potential undiscovered diversification hotspots.

If multiple known species occur on the handful of sky-islands in southeastern Arizona, we expect additional undescribed species on the dozens of montane sky islands in northern Sonora. For many lineages, montane populations in southern Arizona constitute a “northern tip of a southern iceberg”, with centers of distribution found in northern Mexico (e.g., montane jumping spiders - Maddison and McMahon 2000, montane rattlesnakes - Bryson et al. 2011). The desert canyons of southern California plus Baja California Norte constitute another distributional area with potentially high unknown diversity. This region is tectonically active. For example, the population at Mountain Palm Springs lays almost directly on the Elsinore Fault, while the Borrego Palm Canyon population inhabits the area between the San Felipe Fault and the more easterly San Andreas Fault (Steely et al. 2009). Furthermore, relictual canyon populations are expected to be strongly isolated by unsuitable xeric habitats, leading to active speciation. As an example, Bond (2012) described multiple species of cryptic trapdoor spider species from a small region in Anza-Borrego Desert State park, with each species known only from one or two locations. Similar desert canyon local endemics likely occur in *Sitalcina*.

### Historical biogeography

Niche modeling reveals a conspicuous geographic gap between habitats that are suitable for *Sitalcina*, separating eastern habitats in Arizona from western habitats in California (Figure 7). This gap is not only consistent with biotic features that separate these regions (i.e., low elevation Sonoran desert), but also coincides with major modern day and historical biogeographic barriers. These biogeographic barriers include marine incursions into the Salton Trough associated with the opening of the Gulf of California (ca. 6.3 Ma, Oskin and Stock 2003, Dolby et al. 2015), and the more recent drainage of the Colorado River into the Gulf (ca. 4.1 Ma, Dorsey et al 2007, Dolby et al. 2015). Moreover, populations on either side of this biogeographic gap are currently found on different continental plates – all *Sitalcina sura* group populations from California are found on the Pacific plate, while all populations from Arizona are found on the North American plate (we note here that Anza-Borrego canyon populations are close to plate boundaries, further discussed below). For these reasons, a logical prediction would include a primary phylogenetic division in this region, resulting in California versus Arizona clades. Major phylogenetic or phylogeographic splits coincident with the Colorado River (or Salton Trough) have been found in many animal taxa (e.g., Riddle et al 2000, Riddle and Hafner 2006, Crews and Hedin 2006), although we emphasize that these studies have focused mostly on low elevation desert species.

We failed to recover this predicted pattern, but instead recovered a well-supported phylogenetic pattern in both gene and species trees that includes the separation of *Sitalcina sura* group members into coastal versus desert clades (Figures 3, 4, 6). Here, southern Californian populations from the Anza Borrego desert region are phylogenetically allied with Arizonan populations, rather than with geographically proximate Californian populations. Because all lines of evidence indicate that *Sitalcina* is dispersal-limited, we hypothesize that vicariance has dominated the biogeographic history of this lineage. Below we present a biogeographic model for *Sitalcina* that emphasizes the role of plate tectonics and habitat connections to elements of a MTG.

**Figure 13.**
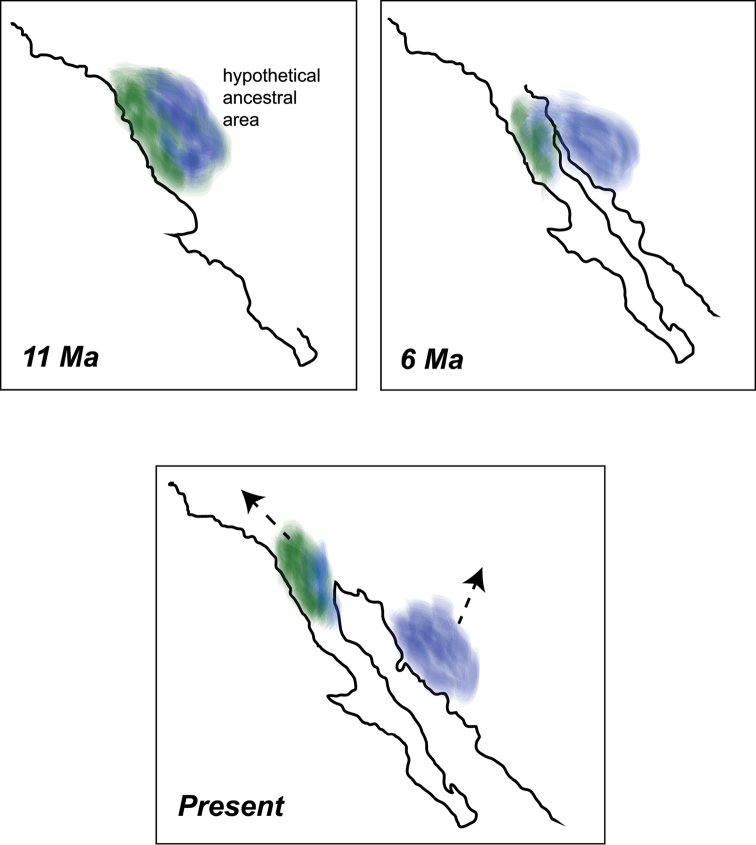
Biogeographic model. General timeframe and plate positions follow Dolby et al. (2015). The labeled “hypothetical ancestral area” is only approximate; arrows indicate present distribution of lineages.

The fundamental spatial premise of our model is that the *Sitalcina sura* group has a center of origin in northwestern Mexico, perhaps centered around the region which is today the northern Gulf of California. The fundamental temporal premise is that initial diversification in the group happened before the Pacific plate began to migrate actively northwestwards (along the San Andreas Fault system) against a stationary North American plate (Figure 13). Geological evidence indicates older rifting beginning as early as ca. 30 Ma in northwestern Mexico, with more active northwestern plate movement sometime after ca. 12.3 Ma (Atwater and Stock 1998, Dolby et al. 2015). Furthermore, our model postulates initial diversification along a west to east axis, with westerly coastal clade members (plus *Sitalcina lobata* and *Sitalcina californica*), and easterly desert clade members. Importantly, this eastern lineage is hypothesized to have straddled both continental plates prior to active plate movements (Figure 13). Our estimates for the overall age of the *Sitalcina sura* group, and for the tMRCA of desert versus coastal clades are generally consistent with this predominantly Miocene timeframe. Also, estimated times for the separation of CA canyon taxa from remaining desert clade members are consistent with the evolution of the Gulf of California (tMRCA of 7.09, Figure 6B, versus ca. 6.3 Ma, Oskin and Stock 2003, Dolby et al. 2015).

Our model has interesting connections to the botanical literature. As noted in the Introduction, most members of the *Sitalcina sura* group (and *Sitalcina* more generally) are found in habitats dominated by sclerophyllous woody plant taxa (e.g., *Quercus*, Pinyon pine, *Arctostaphylos*, *Ceanothus*, etc.). These plant lineages are part of a MTG, as originally defined by Raven and Axelrod (1978). Although this concept was invoked prior to modern phylogenetic analyses, recent molecular studies have generally supported core aspects of this idea (Lancaster and Kay 2013, Baldwin 2014). Important properties of plant lineages included in this flora include southwestern ancestry, deepest divergences occurring in the Oligocene and/or Miocene (e.g., Lancaster and Kay 2013, figure 2), a proposed role for plate tectonics (e.g., Raven and Axelrod 1978, fig. 4), and biogeographic connections between California and Arizona upland taxa (Raven and Axelrod 1978, table 7). These aspects of the MTG are consistent with our biogeographic model. Another interesting parallel is that many Madro-Tertiary taxa now found in coastal southern California are hypothesized phylogenetic relicts, found only in suitable habitats directly adjacent to a marine influence (Raven and Axelrod 1978). In this regard, we note that coastal clade *Sitalcina sura* group species are all found in moist mountains with direct marine influence (e.g., Palos Verdes peninsula, Santa Monica Mountains, Santa Ynez Mountains, Santa Lucia Range), with the single exception of *Sitalcina chalona*.

Several predictions can be derived from our biogeographic model. First, we expect undescribed species in upland habitats of western Sonora, some of which may be related to California desert canyon taxa. Second, we predict undiscovered populations in desert canyons of northern Baja, and again predict possible mixed phylogenetic affinities (some coastal clade, some desert clade). Third, we predict that northern populations of *Sitalcina californica* on the North American plate (north of San Francisco) should be phylogenetically derived from southern populations on the Pacific plate (Figure 1). Finally, if our model has generality, we predict that future studies of other regional dispersal-limited animal lineages will show similar biogeographic patterns.

Both California and deserts of the American southwest are active areas for modern biogeographic research in animals. Despite this fact, we have found relatively few animal taxa that show biogeographic patterns similar to those observed in *Sitalcina*. Salamanders in the *Batrachoseps pacificus* group have a distribution much like the coastal clade (from Monterey south to northern Baja, almost all populations restricted to Pacific plate, Jockusch et al. 2014), and the group includes isolated southern California desert populations, but these populations have apparent western ancestry (Martínez-Solano et al. 2012,). *Aptostichus* trapdoor spiders include many California coastal, California desert canyon, and Arizona sky island species (Bond 2012), but phylogenetic details remain uncertain. One compelling parallel is found in night lizards (*Xantusia*). Leavitt et al. (2007) found that taxa from mostly transmontane southern California and northern Baja (including *Xantusia wigginsi*, “San Jacinto” Clade, and “Yucca Valley” Clade) are sister to *Xantusia bezyi* from uplands of central Arizona. Western species are found in pinyon-juniper and desert chaparral, while eastern *Xantusia bezyi* occur in desert chaparral. These lizard taxa are hypothesized to have diverged 6.4 Ma, coincident with the evolution of the Gulf of California (Leavitt et al. 2007). All of these biogeographic patterns are similar to those observed in the *Sitalcina* desert clade.

## Conclusions

We have uncovered an apparently novel phylogenetic pattern in a biogeographically well-studied region. Our biogeographic model can be tested with additional research, both in *Sitalcina*, but also in other plant and animal lineages with similar geographic distributions. Ubick and Briggs (2008) suggested that *Sitalcina* likely included additional undescribed species – our data support this contention, and suggest even more undiscovered richness. Basically all members of the *Sitalcina sura* group have very small geographic distributions, and many represent apparently old lineages found in often disturbed habitats. Examples include the Palos Verdes population, known only from a single impacted canyon in a matrix of urban development. Similarly, the two known species from Anza-Borrego Desert State Park are each known only from single desert canyons, both located in close proximity to popular hiking trails. These short-range endemic taxa should receive more conservation attention than currently afforded (Harvey 2002, Harvey et al. 2011).

## Supplementary Material

XML Treatment for Sitalcina borregoensis

XML Treatment for Sitalcina oasiensis

XML Treatment for Sitalcina ubicki

## Supplementary materials

Supplementary material 1 Supplementary Tables 1–3 Table S1. Voucher and locality data Table S2. PCR primer and reaction information Table S3. GenBank accession information Data type: Excel Table Explanation note: This dataset is made available under the Open Database License (http://opendatacommons.org/licenses/odbl/1.0/). The Open Database License (ODbL) is a license agreement intended to allow users to freely share, modify, and use this Dataset while maintaining this same freedom for others, provided that the original source and author(s) are credited.Angela DiDomenico, Marshal Hedin
